# Effects of prolonging administration gonadotropin on unexpectedly poor ovarian responders undergoing in vitro fertilization

**DOI:** 10.1186/1477-7827-8-26

**Published:** 2010-03-17

**Authors:** Zhaolian Wei, Xianxia Cheng, Huirong Li, Yunxia Cao, Lin Cong, Ping Zhou, Jun Li

**Affiliations:** 1 Reproductive Medicine Center, The First Affiliated Hospital, Anhui Medical University, Hefei, 230022, PR China; 2 Pharmacological college, Anhui Medical University, Hefei, 230022, PR China

## Abstract

**Background:**

There are still some patients who show poor response to ovarian stimulation prior to evidence of normal ovarian reserve in vitro fertilization. However, there are few studies about how to treat the unexpectedly ovarian poor responder in vitro fertilization. The main aim of this study evaluate the effect of prolonging administration follicle-stimulating hormone in woman with the unexpectedly ovarian poor responder in vitro fertilization on implantation rate, clinical pregnancy rate and live birth rate.

**Methods:**

922 patients subjected to IVF were divided into two groups according to the predicted criterion of ovarian poor response. 116 patients predicted poor response received the short protocol (group C). The others received the long protocol, among the latter, there were 149 patients undergoing unexpectedly ovarian poor response (group B) and 657 patients exhibited normal ovarian response (group A). The doses of gonadotropin, duration of administration, implantation rate, clinical pregnancy rate and live birth rate were recorded among three groups.

**Results:**

The implantation rate of embryo, clinic pregnancy rate and delivery rate are similar between the group A and group B, while there are significant differences between the doses of gonadotropins (35.1 +/- 8.9 ampules vs.53.0 +/- 15.9 ampules) and the duration of administration (15.3 +/- 3.6D vs. 9.8 +/- 2.6D) of these two groups. There are no significant differences about clinical pregnancy rate and live birth rate between group B and group C.

**Conclusion:**

Prolonging administration gonadotropin on the unexpectedly poor ovarian responders does not lower live birth rate in vitro fertilization.

## Background

The success of in vitro fertilization (IVF) depends on careful patient selection and adequate controlled ovarian hyperstimulation. It is estimated that 5%-18% of all IVF cycle are complicated by poor response to ovarian hyperstimulation. Poor response to goandotropin may result in reduction in the pool of embryos available for transfer or cryoperservation, and decrease pregnancy rates. There is still no consensus definition of poor responder [1]. The following criterions had been used to define "poor responders" in practice [2]: No. of mature follicles <2-5; No. of mature oocytes retrieved ≤3; Single dominant follicle; Mean daily gonadoropin dose ≥300 IU; Total gonadotropin dose >40 ampules [3].

It is necessary to identify low responders prior to hormonal treatment for in vitro fertilization-embryo transfer, so that the patients can be counseled regarding the lower chances for pregnancy, more realistic expectations, and cautiously alternative therapies such as oocyte donation or adoption. In addition, stimulation protocols for these patients can be modified to lower the risk of cancellation and improve pregnancy rates. As a result, many screening methods have been proposed to assess prospectively ovarian reserve and individualize treatment regimens. Ovarian reserve decline has been proved to related to age, hormone screening tests, the number of antral follicles, basal ovarian volume, and the result of their previous IVF/HCG treatment.

However, some patients will still be poor responders, although there is no suggestive evidence of low ovarian reserve in IVF [4]. There are few literatures about how to deal with unexpectedly poor ovarian response in order to increase the number of oocytes retrieved, lower cyclical cancellation rate, and improve the IVF result. The aim of this study is to evaluate whether prolonging administration FSH in unexpectedly poor ovarian responders could cut down cancellation rates and attain ideal pregnancy rates.

## Methods

### Study design

This is a retrospective analysis of prolonging administration gonadotropin on unexpectedly ovarian poor responders undergoing fresh IVF/ICSI cycles at the reproductive center of the first affiliated hospital of Anhui medical university. In a review of our electronic database, we found 149 patients who have unexpectedly ovarian poor response during in vitro fertilization between Jan 2005 and Dec 2006. Controls were matched for the data of that included: IVF procedure, age, basal antral follicle count, basal FSH, infertility diagnosis and intracytoplasmic sperm injection (ICSI) or conventional insemination. The analysis was approved by the Institutional Review Board (IRB) of Affiliated Hospital of Anhui Medical University. The main outcome is comprised of the live-birth rate per cycle. Other outcomes are the number of duration of gonadotropin therapy, ampules of gonadotropin, number of oocytes retrieved, clinical pregnancy rates and pregnancy loss rates.

### Patients

The study was carried out between Jan 2005 and Dec 2006 in the reproductive center of the first affiliated hospital of Anhui medical university. A diagnosis of poor responders was made if patients matched the following criteria: age >38 y, serum basal FSH over 10 IU/ml, basal antral follicle counts (BAFC) ≤5. According to the predicted criterion of ovarian poor response, 922 patients subjected to IVF were divided into three groups. 116 patients with predicted poor response received the flare-up GnRH agonist protocol (group C);The others received the GnRH agoinst long protocol. Among the latter, 657 patients were normal ovarian response (group A), however there were 149 patients with unexpectedly ovarian poor response (group B). Here unexpectedly ovarian poor responders were defined using the following criterion [5]: the duration of gonadotropin administration ≥ 14 d and the doses of gonadotropin >40 ampules.

### Ovarian stimulation

Patients in group A and group B received muscular injections of 1.87 mg luteal gonadotropin-releasing hormone (GnRH, leuprolide acetate) in the midluteal phase of the previous cycle. Two weeks later, FSH (Gonal-F, Serono) was administered at an initial dose of 225 IU per day. The FSH dosage was modified according to the ovarian response monitored by serial transvaginal scanning after 5 days. In group C, patients were stimulated with a short protocol using down-regulation by GnRH from day 2 of the cycle (leuprolide acetate 1.87 mg) and muscular injection of FSH (Gonal-F, Serono) at a dose of 225 IU from day 5 of the cycle. The FSH dosage was adjusted according to the ovarian response monitored by serial transvaginal scanning after 7 days of stimulation. Oocyte retrieval was performed by transvaginal aspiration approximately 36-40 h after injection 10 000 IU of human chorionic gonadotrophin (HCG, Lizhu, China) intramuscularly (IM) when two or more follicles reached >18 mm. Insemination take placed by standard IVF or ICSI.

### Embryo transfer technique

Embryo transfers were performed 3 days after oocyte retrieval using a flexible catheter (Wallance, Smiths Medical Internation, UK). The number of embryos transferred was 2-3. The cervical canal was cleaned with warmed culture media before transfer. Luteal support was starting from the day of "ICSI" untill 12 weeks of gestation with 60 mg Progesterone (Xianju, China) by intramuscular injection daily. Urinary HCG test was performed on day 15 after embryo transfer to confirm pregnancy. Clinical pregnancy was determined when a gestation sac was seen by transvaginal ultrasound at 5 weeks after embryo transfer.

### Statistical analysis

The SPSS12.0 package was used for statistical analyses. Data were expressed as means ± SD. Students t and chi-square tests were used when appropriate and P < 0.01 was considered statistically significant.

## Results

### Baseline characteristics of women

Patient characteristics of the two different stimulation protocols are presented in Table 1. The mean ages of group A and group B are similar. There were no noticeable differences in body mass index (BIM) neither, as well as in basal serum FSH and E2, basal antral follicle counts and endometrial thickness on the day of oocytes retrieval between two groups. Basal serum FSH has statistically differences between group A and group C (6.12 ± 2.49 IU/L vs. 7.92 ± 2.88 IU/L, *P *< 0.01).

**Table 1:** Clinical information and basal hormonal profiles in three groups

	A group	B group	C group	*P *value
No. of patients	657	149	116	NS
Age (years)	30.6 ± 3.3	30.6 ± 3.4	38.5 ± 2.1	< 0.01
Body mass index (kg/m^2^)	20.3 ± 1.6	22.3 ± 1.7	21.4 ± 1.5	NS
BAFC	12.5 ± 2.1*	11.6 ± 3.2*	4.4 ± 1.9*	< 0.01
FSH (IU/L)	6.12 ± 2.9	6.72 ± 2.8	7.92 ± 2.9	< 0.05
LH (IU/L)	4.6 ± 3.4	4.76 ± 3.3	4.50 ± 2.3	NS
Estradiol (nmol/L)	169.8 ± 131.2	183.10 ± 162.5	192.4 ± 144.1	< 0.01
Endometrial thickness(mm)	11.8 ± 2.7	11.7 ± 2.6	10.93 ± 4.1	NS
Number of ampoules of Gn	35.1 ± 8.9	53.0 ± 15.9	38.1 ± 16.1	< 0.01
Stimulation duration (days)	11.5 ± 1.2	15.3 ± 3.6	9.8 ± 2.6	< 0.01
Peak E2 (nmol/L)	18672.3 ± 5462.4	16769.2 ± 7685.81	9734.8 ± 1245.5	< 0.01
Oocytes retrieved	16.1 ± 7.9	13.7 ± 9.2	7.6 ± 5.3	< 0.01
Embryos transferred	2.3 ± 0.6	2.3 ± 0.5	2.9 ± 0.7	< 0.01

BAFC: basal antral follicle counts. Serum peak E2 was measured in injection HCG day. Values are reported as mean ± SD unless otherwise stated. *P *< 0.01 was considered statistically significant

### Gonadotrophin administration of three groups

There were significant differences among the doses of gonadotrophins (35.09 ± 8.96 ampules vs. 52.97 ± 15.93 ampules) and the duration of administration (9.78 ± 2.60D vs. 15.34 ± 3.56D, p < 0.01) between group A and group B. In group A, the number of oocytes retrieved was higher than that in group B (16.09 ± 7.95 vs.13.72 ± 9.19, *P *> 0.01). Further more, the doses of gonadotrophins (2.97 ± 15.93 ampules vs. 38.13 ± 16.14 ampules) and the duration of administration (15.34 ± 3.56D vs. 9.78 ± 2.60D) were statistically different between group B and group C (p < 0.01). The number of oocytes retrieved was found to be significantly increased in group B compared with group C (7.57 ± 5.25 vs.13.72 ± 9.19, *P *< 0.01). (Table 1)

### Comparison of treatment outcomes

As summarized in Table 2, clinical pregnancies, embryo cleavage, good embryo quality and implantation rates were comparable in groups A and groups B, but we observed no significant different about embryo implantation (20.8% vs.20.1%, P > 0.01), clinical pregnancies rates (34.3% vs.31.9%, P > 0.01) and live birth rate (23.6% vs. 22.5%) between these two groups. Fertilization and embryo cleavage rates were similar in the group A and group C (P > 0.01), while there were significant differences among good embryo quality rate (89.4% vs. 67.8% P < 0.01), implantation rate (20.8% vs.16.5% P < 0.01) and clinical pregnancy rate(34.3% vs. 21.6%, P < 0.01). But clinical pregnancy rate and live birth rate was comparable for women in groups B and C.

**Table 2:** Comparison of clinical outcome of three groups

	A group (n = 657)	B group (n = 149)	C group (n = 116)	*P *value
Insemination rate (%)	73.3	72.4	79.94	NS
Embryo cleavage rate (%)	90.6	87.2	93.1	NS
Good quality embryo rate (%)	89.4*	86.7	67.8*	< 0.01
Embryo implantation rate (%)	20.8*	20.1	16.5*	< 0.01
Clinical pregnancy rate (%)	34.3*	31.9	21.6*	< 0.01
Abortion rate (%)	19.6	16.9	15.7	NS
Live brith rate (%)	23.6	22.5	15.0	NS

Note: * *P *< 0.01 was considered statistically significant

## Discussion

Our data show that the live-birth rate of prolonging administration FSH on the unexpectedly poor ovarian responders with normal FSH responsiveness during IVF cycles was similar. The results indicated indirectly that administration FSH in long time does not impair oocytes and embryos quality.

Ovarian stimulation protocols aim to enhance follicular recruitment and avoid spontaneous ovulation. Selecting the appropriate ovarian stimulation protocol and controlling the dose of gonadotrophin, not only can obtain the best curative effect but also reduce the risk of poor ovarian response and ovarian hyperstimulation syndrome. Assessment of ovarian reserve in women undergoing assisted reproduction is useful in optimizing the treatment protocol and in counselling the patients. Poor responder was predicted by the presence of the following characteristics [6]: age >35 y; basal serum FSH level (bFSH ) >8 IU/ml, or FSH/LH ≥3; the basal volume of each ovary <3 ml, the number of antral follicle <6; basal serum inhibin B level <45 pg/ml. In assisted reproduction programs, the response of "good responders" or "poor responders" to exogenous FSH is individualized and the ovarian response to intense gonadotrophin stimulation is difficult to predict. The response of some patients will be poor though their predictive tests were not being suggest low ovarian reserve. The sensitivities varied for the prediction of poor ovarian response varied between 39% and 97%, and specificities between 50% and 97% [7]. At present, we can't accurately predict poor response in IVF/ICSI cycles, there are still 16.16% cases in unexpectedly poor ovarian response in our center.

Unexpectedly poor responders represent a heterogeneous group of patients. Different mechanisms have been proposed to explain poor ovarian response such as decreased number of FSH receptors in granulosa cells [8], defective signal transduction after FSH-receptor binding [9], anti-FSH IgA and IgG potentially exerting a local FSH antagonizing effect in maturing follicles [10], the presence of a specific FSH receptor-binding inhibitor in the follicular fluid [11], the higher FSH threshold to stimulation follicle development.

The most prevalent approaches for treating poor responder are always the GnRH agonist flare protocol. Compared to traditional long GnRH agonist protocol, there is no significant difference in clinical pregnancy rates [12,13]. Our data showed that increased doses of gonadotropins were not able to influence ovarian response in poor responders (group C), the pregnancy rate for poor-responder patients and number of oocytes retrieved are lower than the two other groups (group A and group B).

GnRH antagonist may shorten the duration of stimulation, lower the total gonadotropin requirements, reduce patient's cost, decrease cycle cancellation, and has a higher ongoing pregnancy and a better delivery rate in poor responder patients [14,15]. There is a new method to use GnRH antagonist before ovarian stimulation in order to lengthen the follicle phase and rescue more follicles. Doing so, more oocytes and zygotes were attained, implantation rate and ongoing pregnancy rates were improved [16]. Addition of LH may be beneficial effect in poor ovarian responders [17,18], but other prospective and randomized trial show that the addition of rLH to the ovarian stimulation protocol produces no further benefit in older poor responder patients [19]. Endogenous FSH in the preceding luteal phase can stimulate larger follicles and subsequently lead to a size discrepancy. A novel strategy for treating poor responders is to give estradiol in the luteal phase before IVF hyperstimulation, the estradiol can suppress FSH in the preceding luteal phase and result in more coordinated cohort of follicles responding to the stimulation process. The luteal E_2 _protocol may improve embryo quality and delivery rates [20-22]. There is few study about treatment on the women in unexpectedly poor ovarian responders in IVF cycle. Pretreatment with transdermal testosterone may improve ovarian response to gonadotrophins and ovarian sensitivity to FSH in low-responder IVF patients with normal basal concentration of FSH and in previous low- responder IVF patients [23,24]. Our study showed that prolonging to administration FSH on the unexpectedly poor ovarian responders only reflect a decreased number of oocytes retrieved, But clinical pregnancy loss rate and live birth rate were simliar between group A and group B in the study. The result is accordance with other reports [25]. Prolonging to administration FSH on the unexpectedly poor ovarian responders could raise financial burden to couples, but reduce the psychological distress related to cycle cancellation.

## Conclusions

Prolonging administration FSH on the unexpectedly poor ovarian responders could increase number of oocytes retrieved, reduce number of cancelled cycles and improve IVF outcomes. Future studies would be helpful to reveal the mechanism so that stimulation protocols for these patients can be modified to lower the risk of cancellation and improve pregnancy rates.

## Competing interests

The authors declare that they have no competing interests.

## Authors' contributions

XXC carried out the statistical analysis. HRL participated in the sequence alignment and drafted the manuscript. LC and PZ collected materials. YXC and JL participated in its design and coordination and helped to draft the manuscript. All authors read and approved the final manuscript.
